# Exploring the evolution and future prospects of Amharic to English machine translation: a systematic review

**DOI:** 10.3389/frai.2025.1456245

**Published:** 2025-05-23

**Authors:** Muluken Hussen Asebel, Shimelis Getu Assefa, Mesfin Abebe Haile

**Affiliations:** ^1^Department of Computer Science and Engineering, School of Electrical Engineering and Computing, Adama Science and Technology University, Adama, Ethiopia; ^2^Department of Research Methods and Information Science, Morgridge College of Education, University of Denver, Denver, CO, United States

**Keywords:** machine translation, Amharic, English, systematic review, low-resource languages

## Abstract

**Introduction:**

In the last couple of decades, Amharic-English translation has greatly improved from a rule-based approach to contemporary systems that apply neural networks. Even after these advancements, problems remain because of the Amharic language’s resource-scarce nature, such as inadequate datasets, tools for working with the language, and the intricate semantics and grammar of Amharic as compared to English. This systematic review seeks to analyze the evolution of the Amharic-English machine translation, the prominent ongoing difficulties, the noteworthy research undertakings, and the prospects of the research focus.

**Methods:**

This review uses a systematic approach to study the literature on Amharic-English machine translation. Important documents were retrieved from academic websites, and those with relevance to the methodologies of machine translation, language resources development, and evaluation practices were chosen. Primarily, the focus was on both statistical and neural machine translation models, especially those with transformer structures.

**Results:**

The initial attempts to translate English to Amharic and vice-versa relied on statistic machine translation (SMT), which set the stage for the evolution to neural machine translation (NMT). The use of transformer models has impacted the accuracy and fluidity of translations tremendously. Still, there is a lack of sufficient parallel corpora, effective methods for tokenization of Amharic, and other resources. Recently, the focus has been on creating new datasets, improving token-level engineering, and modifying NMT models for Amharic’s complex morphological structure.

**Discussion:**

The complete solutions for enhancing Amharic-English translation remain elusive and include the lack of sufficient data, semantic correspondence, and grammatical consistency within and across translations. Pursuable avenues include augmentation of data, tokenization on the language level, and incorporation of linguistic elements into the parallel corpora. In addition, creating effective evaluation frameworks along with comprehensive linguistic data is important for assessing and improving translation tools. With these changes, cross-cultural interaction and increasing accessibility to modern technologies will be achieved.

## 1 Introduction

Amharic is a Semitic language primarily spoken in Ethiopia, where it serves as the official working language of the government (Gebremedhin, 2014; Macro, 2006). It is the second-most spoken Semitic language in the world after Arabic, with over 32 million native speakers and millions more using it as a second language (Teshome and Besacier, 2012). Amharic holds a central place in Ethiopian society, culture, and education. It is the language of instruction in schools, a medium for government communications, and a key vehicle for national literature and media.

Amharic is inscribed in the Ge’ez script, which is distinct from Latin alphabet in that it possesses its own set of symbols and syllable structure (Teshome and Besacier, 2012). The Ge’ez script, in combination with the morphologically rich and highly inflectional and derivational nature of Amharic, makes the language more complex. On top of that, Amharic as a language of wide communication has a complex oral heritage comprised of narrative folktales, cultural proverbs, and verse literature which helps to express community norms and ethics as well as history. This depth of culture and uniqueness in language makes Amharic of indisputable historical and cultural importance within and outside the country of Ethiopia.

The shift from Amharic to English Machine Translation (MT) has gained significance because of its potential for facilitating communication and bridging the gap in various fields (Melese et al., 2017). MT makes it easier for people, firms, and institutions to get information, collaborate, and interact with a wider audience without the dependence on manual translations (Melese et al., 2017). This type of MT aids in fostering understanding across different cultures, knowledge transfer, and global linking (Shadiev et al., 2019). Furthermore, it can assist in transferring Amharic material to English speakers and the other way round, thus aiding in cultural exchange and inclusiveness. Enhancing MT for Amharic has wider repercussions in the area of computational linguistics. The complex morphology and syntax of Amharic is a challenge, but it also presents an opportunity for devising novel methods and systems in natural language processing (Nigusie, 2024). Progress in this area would also improve the effectiveness of MT systems for other morphologically complex, low-resource languages, thus increasing the diversity and equity of language technologies internationally.

Review studies have revealed the historical development of machine translation, showing major advancements and new developments in the discipline. These studies point out that low-resource languages represent a major challenge in natural language processing and machine translation due to data scarcity and language heterogeneity. The lack of sufficient parallel and variated datasets impedes the construction of efficient translation and comprehension models. Research on adapting large language models (LLMs) for these languages highlights the need for parallel data during pretraining and also fine-tuning, suffering performance in its absence (Iyer et al., 2024). Furthermore, the complex nature of morphologically rich languages, dialects and some Creoles makes the situation more challenging, so that traditional natural language processing (NLP) techniques are not applicable (Nzeyimana, 2024). Rivera-Trigueros (2022) sought to determine the most viral machine translation systems by looking for their architectural, quality control features, and the systems that provide it. Most importantly, this investigate highlights machine translation’s crucial contribution toward addressing language challenges and promoting multilingual information accessibility. This research is focused on the English–Spanish language pair and does not make predictions for the future.

To solve the problem of translating low-resource languages, researchers have employed methods such as backtranslation, data augmentation, fine-tuning, and other forms of transfer learning. These techniques have shown amazing results in languages translation, for instance, in Kannada, Lao-Vietnamese and Kinyarwanda, there has been many increases in BLEU scores (Prasada and Rao, 2024; Tran, 2024). These improvements point out the possibilities of adaptive training methods, as well as tailored approaches to alleviate the problem caused by inadequate data.

Even though Neural Machine Translation (NMT) systems tend to outperform traditional methods, they still have some challenges to solve with regards to low resource situations. For instance, Bangla-English and some Arabic dialects suffer due to high linguistic variability and poorly designed datasets, which leads to very bad NMT results (Abdul-Nabi et al., 2024). However, there is good hope that the problem will be solved by attention-based hybrid models and hierarchical learning frameworks.

If progress is made toward NLP support for underrepresented linguistic cultures, the innovation and collaboration gap in low-resource languages will start to close. It will be necessary to devise methods for faster resource gathering, create synthetic data, follow more effective routes for transfer learning, and cross different linguistic borders share knowledge. Overall, such approaches will reflect on increasing access to these technologies for less represented cultures, hence improving cultural diversity. The phrase “low-resource languages” introduces an issue that is multi-dimensional in its iteration which requires technology improvements, strategic step data solutions, and collaborative work along with studying lexicons that provide the possibility of pattern identification (Joshi et al., 2024). Focusing on these challenges will make offering NLP systems and services, with a primary focus on MT, feasible for underrepresented speech communities, which will assist in fostering equal opportunity Language Technology global access.

The primary focus of this systematic review is to analyze the development over time and forecast the future of Amharic-to-English translation using machines. This review concludes by integrating existing evidence and analyzing crucial trends in the field’s evolution, current status, and potential developments. The specific goals of the review are as follows:

1. *Document historical developments and milestones*: the Amharic to English MT retrospective begins with some of the industry’s greatest achievements, technological progress, and its fundamentals. As well, there is consideration of the shift from traditional systems based on neural networks in contemporary times.
2. *Identifying key challenges and limitations*: ideally, it is to think about the profound problems and gaps observed in the older and more recent Amharic to English MT systems. This incorporates technical, linguistic, and methodological stagnation and most of the fundamental innovations needed.
3. *Assess current trends and future directions*: to summarize the last activities and newly emerging designs in Amharic to English MT. This involves new drivers of the discipline: technologies, methodologies, and new partnerships. It also analyzes and predicts what future research initiatives and potential results will be in the artificial intelligence and machine translation fields with regard to improving the quality and effectiveness of the systems.

This review intends to add to the scholarly and practical perspectives on English and Amharic machine translation, in view of its uses for researchers, developers, and even policymakers who are concerned with the implementation and development of machine translation MT systems is hoping. This has been achieved in conjunction with the predetermined objectives of the review.

## 2 Rationale for the study

To begin with, it is important to highlight the significance of studying the Amharic-English machine translation (MT) language pair. The technological improvement of machine translation has not changed the use of many English and Amharic languages because the syntactic structure and order of these languages are so different from each other. Amharic, a low resource language with rich morphology and little parallel corpora is greatly affect the translation system’s precision and effectiveness.

In earlier times, the discipline exchanged rule-driven systems for neural ones in which asynchronously trained linear networks were used for which translation has gotten better but still does not fully resolve problems. Also, scarcity of data and difference in syntax is still challenge to the performance of the model. In Amharic and English machine translation, this review’s contribution The interventions were focused on the increase of parallel corpora, improvement of tokenization, and application of linguistic knowledge that have been aimed at improving the machine translation focused on enabling the specific issues.

## 3 Intended audience

The most important audience for this study involves the stakeholders from NLP and MT practitioners and researchers. This category comprises people involved in the optimization of MT systems, especially for the less widely spoken languages. The study also caters to some computational linguists, data analysts, and developers of language technologies who deal with the interface of linguistic resources with MT systems.

These stakeholders include decision makers from language and culture preservation organizations, cross-culture communication, and technology practitioners. These stakeholders need tools and technologies that can facilitate and integrate different cultures and languages using sophisticated translation methods. This study provides an in-depth analysis of the different phases of automation of translation from and to Amharic, describes the existing obstacles and tendencies, and offers suggestions for subsequent efforts. The study attempts to contribute to the development of MT and NLP and, at the same time, helps to address the problem of using language and communication technologies for development and integration into the Ethiopian economy.

## 4 Evolution and future prospects of machine translation

### 4.1 Evolution of machine translation

The process of machine translation has become much simpler, owing to the progress in translation aids technologies. We have references systems, then statistical systems, and now with the development of the neural net we move closer to human level communication. There are still problems, like working with low resource languages or providing accuracy in particular fields of expertise, but it feels like every year the technological progress makes it easier and easier to use machine translation. The more we dive into automatic interpretation of languages, the more modest we need to be about our goals of removing boundaries and raise communications as well as understanding between nations. The middle of 2010s was a real turning point thanks to the introduction of NMT during the era of neural networks (Ragni and Nunes Vieira, 2022).

NMT models or the Neural Machine translation models, such as the seq2seq attention model invented by Google Brain, apply deep learning to the translation process by including speech recognition and analysis of sentences as units in the system. These are known to significantly improve over former methods due to their ability to take advantage of long-range dependencies and semantics (Sutskever et al., 2014). NMT has always been a positive approach compared to other methods because it assures production of fluent, precise and context conscious translations. The introduction of NMT has indeed enhanced the quality of machine translation and has made it a core utility for machine translation services, language education, and international business communications.

Machine translation systems are developed based on abundant parallel text data and need significant computing power for training. In comparison, pre-trained models are known to better generalize to new, untapped text while producing more human-like and fluent translations. The advancement of transformer model such as OpenAI’s GPT (Radford et al., 2018) and Google’s BERT (Devlin et al., 2018) has set new limits of the Neural Machine translation with context sensitive and intelligent translations. The progress made in automation has resulted into the revolutionary development in the field of computational linguistics and machine intelligence.

The evolution of Machine Translation (MT) through the years, starting from rule based systems to neural networks, has indeed improved cross linguistic communication. As the research in MT continues to propagate alongside advanced technologies, so does the optimism for achieving understanding through AI powered global Machine Translation. There are miles to go in the process of MT advancement, its techno-socio-centric development will definitely matter toward the future world.

### 4.2 Future prospects of machine translation

Automatic translation is not without its pitfalls. There are still the ever-complex issues of anthropological tendencies sometimes, MT operates in cultures and languages differently from how human beings normally operate. Low-resourced languages that have scarce training data can also be a problem (Koehn and Knowles, 2017). Addressing these challenges requires ongoing research in areas like transfer learning where translations from high resource languages are used to processes low resource languages, and unsupervised learning where the monolingual data that needs to be a processed a NT can be (Artetxe et al., 2019).

Machine translation usefulness of technology will continue evolving with time. Recent developments within Artificial Intelligence and Natural Language Processing continues steggan widespread innovation, even more precise, context insensitive, as well as culturally inclined translation systems seem unavoidable (Castilho and Knowles, 2024). Machine Translation combined with other AI domains like speech recognition and generation systems would make real-time communication across multiple languages more feasible (Woldeyohannis et al., 2018).

## 5 Research questions

This systematic review is structured around three key research questions that guide the exploration of Amharic-to-English machine translation. These questions are designed to uncover historical developments, identify challenges, and explore future prospects in the field:

*RQ1:* What are the historical developments and milestones in the field of Amharic-to-English machine translation?

*Motivation:* this question aims to trace the evolution of Amharic to English MT, highlighting significant projects, technological advancements, and key contributions over time.

*RQ2:* What are the key challenges and limitations associated with past approaches to Amharic-to-English machine translation?

*Motivation:* this question seeks to identify and analyze the primary obstacles that have hindered the development and accuracy of MT systems for this language pair, including technical, linguistic, and methodological challenges.

*RQ3:* What are the current trends and future directions in English-language machine translation research in Amharic?

*Motivation:* this question aims to review the latest advancements and emerging trends in the field; explore new technologies, methodologies, and collaborative efforts; and predict future research directions and potential breakthroughs.

## 6 Methodology

We conducted this study using the systematic literature review (SLR) methodology for data retrieval. The SLR process is defined as the methodical and systematic approach of locating primary studies to develop and evaluate a certain research question. Average literature surveys usually do not require as much care to detail when presenting data within a systematized review. A systematic review aimed at combining and summarizing all information concerning a research issue offers greater validity in the conclusions than individual studies. The results are reported based on the latest PRISMA (preferred reporting items for systematic reviews and meta-analyses) framework (Antoniou et al., 2021).

### 6.1 Eligibility criteria

To capture the full evolution of Amharic-English machine translation, we included research from any year of publication. Titles, keywords, and abstracts of papers that met our particular inclusion and exclusion criteria were evaluated for relevance.

*Inclusion criteria:*

- Research papers published in peer-reviewed journals and conference proceedings.
- Studies focusing on machine translation for Amharic and English.
- Research reported in English.
- Only full-text articles were considered.
- Studies from any publication year were included.

*Exclusion criteria:*

- Research on machine translation between Amharic and English that did not address other languages.
- Research work that does not describe Amharic machine translation
- Research work other than machine translation
- Review studies, abstracts, commentaries, posters (short papers), or editorials.
- Articles that were not fully accessible.
- Duplicate articles.

### 6.2 Source of information

To identify relevant publications, we conducted comprehensive searches across five widely used databases and libraries: ScienceDirect,^1^ Google Scholar,^2^ ACM Digital Library,^3^ IEEEXplore,^4^ and SpringerLink.^5^ This approach ensured a focus on reputable and high-quality sources. Our search included examining titles, keywords, and abstracts.

### 6.3 Search strategies

We conducted a thorough search for published literature using the specified resources and search terms. We included all relevant literature without any limitations on publication dates, except for journals from the social science fields. After numerous iterations of trial and error, the final search query is as follows:

[(“Machine translation” OR MT OR “Computer translation” OR “Automatic translation” OR “Automatic text conversion”) AND (“Amharic text” OR “Amharic translation” OR “Amharic text translation” OR “Amharic”) AND (“English language” OR English)].

Table 1 summarizes the results from various academic databases regarding machine translation, specifically its application to Amharic and English. In addition, it provides a clear overview of the literature available across different databases on the topic of machine translation between Amharic and English.

**Table 1:** Keywords used for each database.

Database	Keywords	Search results	Included in the review
Science direct (ScienceDirect)	(“Machine translation” OR MT OR “Computer translation” OR “Automatic translation”) AND (“Amharic text” OR “Amharic”) AND (“English language” OR English)	40	0
Association for Computing Machinery (ACM) Digital Library	+(“Machine translation” “automated translation” “automatic translation”) + (“Amharic”) + (“English”)	37	1
Institute of Electrical and Electronics Engineers (IEEE) Xplore	(“Machine translation” OR MT OR “Computer translation” OR “Automatic translation”) AND (“Amharic text” OR “Amharic”) AND (“English language” OR English)	9	5
Google Scholar	““Machine translation” “Machine translation” OR MT OR “Computer translation” OR “Automatic translation” OR “Amharic text” OR Amharic OR “English language” OR English “Amharic AND English””	96	24
Springer link	(“Machine translation” OR MT OR “Computer translation” OR “Automatic translation”) AND (“Amharic text” OR “Amharic”) AND (“English language” OR English)	19	1

### 6.4 Selection process

The publication selection process involves applying inclusion and exclusion criteria to identify primary sources relevant to our research questions. Specifically, we select research papers, conference proceedings, and book chapters that focus on machine translation between Amharic and English. Any other publications not related to Amharic machine translation were excluded. After finalizing the selection, we compile all the chosen papers from the five search engines into a single CSV file.

### 6.5 Data collection process

We initiated the data collection process by identifying relevant databases and selecting appropriate keywords for our search. The chosen databases included well-known academic resources such as Google Scholar, IEEE Xplore, ACM Digital Library, SpringerLink, and Scopus. Using a set of predefined keywords related to Amharic and English machine translation, we conducted comprehensive searches within these databases.

Upon obtaining the search results, we meticulously examined the titles of the retrieved works to determine their relevance to our research objectives. This initial screening helped us filter out irrelevant papers and focus on those directly addressing machine translation between English and English. Through this systematic approach, we collected a robust set of research works for further analysis.

Next, we applied our inclusion and exclusion criteria to further refine the selection process, ensuring that only pertinent studies were considered. The final step involved consolidating all the selected papers from the different databases into a single CSV file, facilitating organized and efficient data handling for subsequent analysis stages.

### 6.6 Study selection

**Figure 1:**
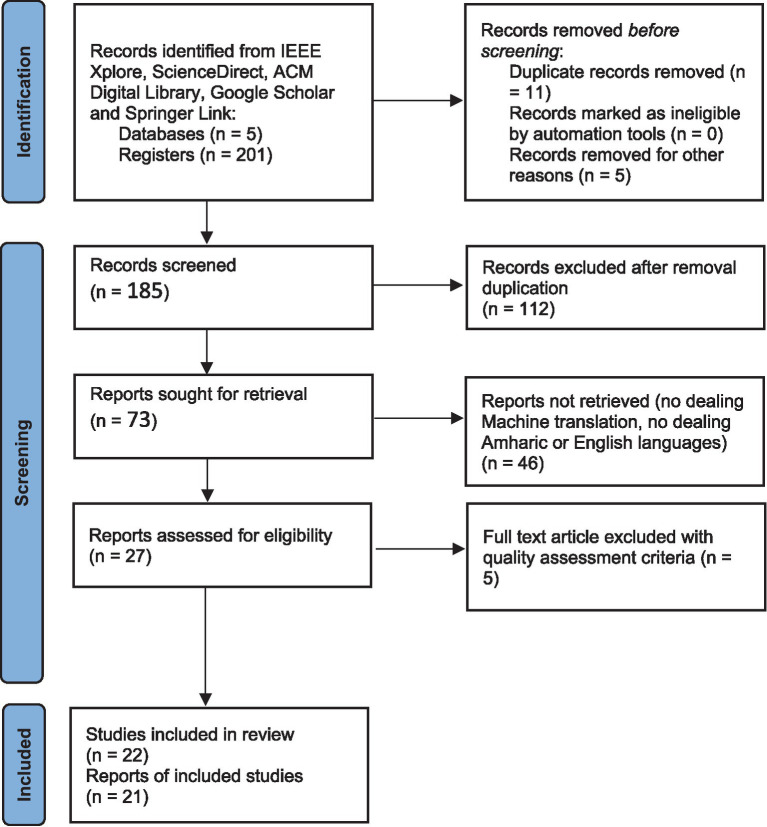
The PRISMA framework for research screening process.

We employed broad searching strategies and selection criteria to retrieve 201 studies from different databases. The article distribution is as follows: ScienceDirect had 40, ACM Digital Library 37. There were 9 in IEEE Xplore, 96 in Google Scholar, and 19 in Springer Link which were all based on our set search words. While following these steps, we collected a lot of records that we later purged of duplicates, bringing the number down to 22. Every single record was then screened against the set inclusion and exclusion criteria to determine their suitability with our research goals. The complete PRISMA 2020 framework is given in Figure 1, which shows the flow of information through the different phases of selection focusing on the eligibility criteria.

These steps guarantee the set of articles left after this process are suitable to form a basis for research on machine translation between English and English.

### 6.7 Data extraction

Pulling out relevant data is pertinent to our systematic literature review because it aids in fetching relevant details from chosen studies in a structured manner. In order to meet this objective, we pilot tested it with a subset of studies and made adjustments as necessary based on the feedback received. Two reviewers independently withdrew the information to reduce bias and any bias was solved by a third reviewer. The information was then verified to be correct, followed by categorization and coding based on previously set criteria about our research questions (RQ1–RQ3). We ensured high quality and reliable information by managing and analyzing the data using tools such as Microsoft Excel. This allows us to answer questions concerning the trends and timely developments in Amharic-English machine translation.

In the data extraction phase, we systematically collected pertinent data from the chosen studies and selected 22 articles based on certain criteria. The gathered data had the following attributes listed below:

- Article type: we accepted published original research, review articles and conference papers while rejecting published reports, case studies and lecture notes.
- Language and domain: the articles had to be in English and from the field of computer science.
- Year of publication: for the purpose of this study, we accepted articles from any year to examine the entire body of work on machine translation between English and Amharic.
- Coverage: the review was not geographically limited and therefore included articles from all countries.

### 6.8 Quality assessment

The assessment phase is equally as important as any other step for our systematic literature review, as it guarantees that the studies incorporated into our review are of the highest quality in terms of rigor, methodology, and validity. We created a set of rules that are drawn out of well-known methodological guidelines to determine the quality of the chosen research papers. Those range from study design to reporting and account for the sample size, data collection techniques, analysis methods, and reporting clarity and coherence. Every study was rated using these criteria through a points based system which differed in the number of points that could be given for criteria such as articulation of research aims, appropriateness and rigor of design, sample size, reliability of instrument of data collection, robustness of methodology for data analysis, and result transparency.

To improve the reliability of the quality assessment, two independent reviewers checked each study individually based on the criteria and scoring system. For disagreements, a third reviewer made the decision for the group and consensus was reached through discussion. This helped maintain an unbiased and systematic evaluation. A score was given to each study assigning a quality category that had the options of high, moderate, or low quality.

In order for our conclusions to be reliable and effective, poorly rated studies were omitted from the analysis. The quality evaluation results were shared and outlined within the report, giving a detailed explanation of the rationales behind inclusion and exclusion for each study. We achieved guarantee for the quality and authenticity of our systematic review on the Amharic-English machine translation’s evolution and future prospects by following these steps.

### 6.9 Data analysis and synthesis

Our systematic literature review employed a particular method for the analysis and synthesis of the gathered data. Using office tools like Microsoft Excel, we sorted the extracted data files into folders. In this case, we organized the data according to predefined themes and variables regarding Amharic-English machine translation. Methods, evaluation metrics, and key findings were identified in this step. We then performed an informative analysis where we described the general characteristics of the studies selected to be included. We studied the distribution by publication year and the type of study conducted, whether original, review or conference papers. This step was useful in identifying the development and changes that have occurred over time in regard to Amharic-English machine translation research.

Once the descriptive analysis was done, we then began with the thematic analysis to search for common patterns or recurring ideas within those studies. We coded the data files to encompass the main concepts and techniques pertaining to Amharic-English machine translation. Themes such as the creation and use of translation models, their evaluation approaches, and difficulties associated with low-resource language translation were identified and analyzed. Moreover, we did a comparative analysis to compare the differences and similarities of the studies. The performance of different automated translation methods and their corresponding measure values as well as the environments where they were deployed was analyzed and compared.

In combining the findings of various studies, we were able to give a detailed description of the existing work on machine translation of Amharic and English. Thematic and comparative analyses were conducted separately, but their outcomes were synthesized to provide insights into the development, problems, and possible changes within this domain in the future. So that our findings could be communicated more easily, we constructed graphs and tables to represent them visually. These visuals highlight key trends, patterns, and comparisons across the studies. Our approach to analyzing the data and how it was synthesized enabled us to develop clear cut answers on the integration and future development of Amharic and English machine translation.

### 6.10 Basic terminology

In this section, we explain fundamental concepts necessary for the discussion of the topic. Amharic, a Semitic language from Ethiopia (Demeke, 2001), and English, a West Germanic language, provide the linguistic context (Bech and Walkden, 2016). Automated translation of text or speech across cultures is achieved through machine translation, which uses computer algorithms to translate (Kembaren et al., 2024). The term “evolution” identifies the processes of change within translation systems over time, while future prospects refer to expected improvements arising from innovations and the use of Artificial Intelligence in machine translation from Amharic into English. This domain, as we know, is the subject of literature and, by definition, a systematic review will cover nutshell all of them (Snyder, 2019).

## 7 Results

### 7.1 Document historical development and milestones

**Figure 2:**
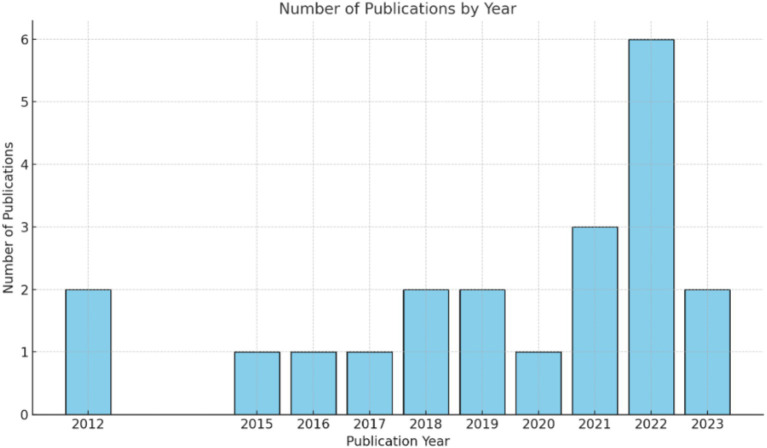
Number of studies per year of publication.

The publication timeline for Amharic to English machine translation spans from 2012 to 2023, as depicted in Figure 2. The inaugural publication emerged in 2012, marking the beginning of scholarly interest in this field. Over the years, there has been a noticeable fluctuation in the number of publications. Certain years stand out with a higher volume of research output, indicating periods of intensified academic focus and advancements. The most significant surge in publications occurred in 2022, which recorded the highest count within the given timeframe. This trend reflects the growing interest and development in the domain of machine translation between Amharic and English. The overall publication pattern exhibits variability, with some years experiencing a greater influx of studies than others do, highlighting the evolving nature of research activity in this area.

**Figure 3:**
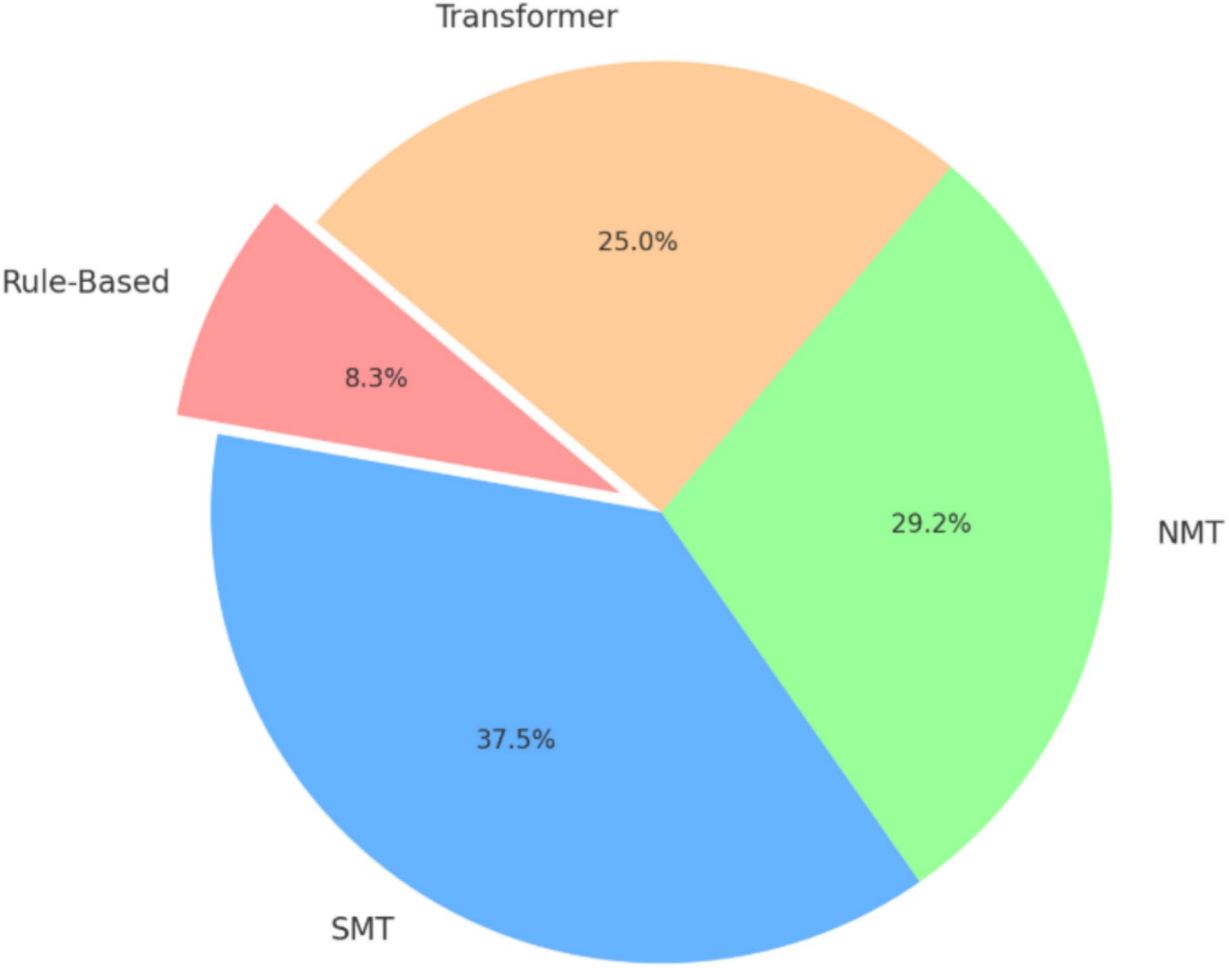
Distribution of methodologies in machine translation research.

Figure 3 illustrates the distribution of machine translation methodologies in percent. These methodologies include Rule-Based Translation, Statistical Machine Translation (SMT), Neural Machine Translation (NMT), and the Transformer model.

- *Rule-based translation (10%):* only two studies employed rule-based approaches, highlighting their limited use compared to modern techniques. De Pauw et al. (2012) and Kore and Goyal (2017) focused on implementing rule-based systems for translating English to Amharic and proper noun transliteration, respectively.
- *Statistical machine translation (SMT, 45%):* SMT dominated earlier research, with nine studies exploring its application for translating between Amharic and English languages. SMT experiments often served as baselines for comparison with NMT methods.
- *Neural machine translation (NMT, 35%):* seven studies utilized NMT, reflecting a shift toward neural approaches. These works included developing attention-based architectures (Gashaw and Shashirekha, 2019), leveraging subwords for handling inflectional morphology (Gezmu et al., 2021), and hybrid methods combining contextual information (Ashengo et al., 2021).
- *Transformer models (30%):* the adoption of Transformers is growing, with six studies emphasizing their effectiveness in low-resource language pairs. Researchers like Hadgu et al. (2022) and Destaw Belay et al. (2022) demonstrated superior performance compared to previous methodologies, using techniques like homophone normalization and corpus augmentation.

The selected publication contains detailed information on various research papers focused on machine translation involving Ethiopian languages such as Amharic, Tigrinya, and Ge′ez (which have the same scripture). Table 2 is organized into columns of authors, methodologies, and contributions. Each entry lists the authors of the paper; the specific machine translation methodology used (including rule-based, statistical, neural, and transformer-based approaches); and a summary of the research contributions. For example, studies by Teshome M. et al. (2015) explored statistical machine translation (SMT) between English and Amharic, achieving incremental improvements in BLEU scores through different experimental setups. Other studies by Gashaw and Shashirekha (2019) examined neural machine translation (NMT) between Amharic and Arabic, comparing LSTM and GRU models. Some works, like Gezmu et al. (2022), focus on creation and enhancement of parallel corpora specifically aimed at improving SMT and NMT models using a large Amharic-English corpus, while other, Hadgu et al. (2020), developed the Lesan translation system which aids low-resource language translation by using transformer models and back-translation methods. Those enabled and insightful dataset reflects the most important milestones, issues, and prospects of machine translation research and development for Ethiopian languages, demonstrating different methods and notable progress in translation quality.

**Table 2:** Key contributions and methodologies used.

Author	Methodology	Contribution
De Pauw et al. (2012)	Rule-based machine translation	Described key aspects of an ongoing project to implement a rule-based English-to-Amharic and Amharic-to-English machine translation system.
Teshome and Besacier (2012)	Statistical machine translation	Discussed the experiment conducted to translate from English to Amharic using the Statistical Machine Translation (EASMT) approach.
Teshome M. et al. (2015)	Statistical machine translation	Focused on improving translation quality by applying phonemic transcription on the target side, resulting in a BLEU score improvement.
Tedla and Yamamoto (2016)	Statistical machine translation	Presented initial research on English-to-Tigrinya SMT, addressing morphological segmentation of Tigrinya words to reduce data sparseness and improve translation quality.
Kore and Goyal (2017)	Rule-based machine translation	Proposed a rule-based machine transliteration technique for English to Amharic proper nouns, achieving 90.08% precision in correct transliterations.
Abate et al. (2018)	Statistical machine translation	Described the development of parallel corpora for English and Ethiopian Languages for bidirectional SMT experiments, highlighting the impact of morphological richness on SMT performance.
Gashaw and Shashirekha (2019)	Neural Machine Translation	Developed Amharic-Arabic NMT models using Attention-based Encoder-Decoder architecture, comparing LSTM and GRU models, and found that LSTM outperforms GRU and Google Translation system.
Abate et al. (2019)	SMT	Described the development of parallel corpora for English and Ethiopian Languages for bidirectional SMT experiments, highlighting the impact of morphological richness on SMT performance.
Gashaw and Shashirekha (2020)	Neural Machine Translation	Constructed a small parallel Quranic text corpus for Amharic-Arabic NMT experiments, comparing LSTM and GRU based models with Google Translation.
Gezmu et al. (2021)	Neural Machine Translation	Described neural machine translation between Amharic and English using a new transliteration technique for Amharic and subwords to handle highly inflectional morphology.
Biadgligne and Smaïli (2022)	SMT and NMT	Developed an English-Amharic parallel corpus and conducted SMT and NMT experiments, with the corpus freely shared for research. SMT achieved 26.47 BLEU and NMT achieved 32.44 BLEU.
Ashengo et al. (2021)	Neural Machine Translation	Investigated a new approach combining context-based machine translation (CBMT) with RNNMT for English-Amharic translation, showing performance improvement over simple NMT.
Biadgligne and Smaïli (2022)	SMT and NMT	Investigated the effect of corpus augmentation on English-Amharic MT quality, showing improved BLEU scores for both SMT and NMT models.
Hadgu et al. (2022)	Transformer	Presented Lesan, an MT system for low-resource languages using a custom OCR system and Transformer model, outperforming Google Translate and Microsoft Translator for Tigrinya, Amharic, and English translations.
Destaw Belay et al. (2022)	Transformer	Developed bidirectional Amharic-English NMT models using the Facebook M2M100 pretrained model, achieving BLEU scores of 37.79 for Amharic-English and 32.74 for English-Amharic translation, and explored the effects of Amharic homophone normalization.
Biadgligne and Smaïli (2022)	Transformer	Applied corpus transliteration and augmentation techniques to improve English-Amharic MT performance, achieving the highest BLEU score for the language pairs using Transformer models.
Gezmu (2023)	morpheme-based NMT	Investigated morpheme-based NMT models for low-resource fusion languages, showing that morpheme-based models outperform conventional subword models on benchmark datasets.
Getachew and Yayeh (2023)	Transformer	Proposed a bidirectional NMT model for Ge′ez-English translation using the Transformer model, achieving BLEU scores of 27.19 for English-Ge′ez and 29.39 for Ge′ez-English translation, despite dataset scarcity.
Gezmu et al. (2022)	SMT and NMT	Described the acquisition, preprocessing, segmentation, and alignment of an Amharic-English parallel corpus, demonstrating that NMT models outperform SMT models by approximately six to seven BLEU points.
Negia et al. (2023)	Transformer	Attempted to design Amharic-Kistanigna bidirectional MT using various deep learning models, concluding that the Transformer model achieved the highest BLEU scores of 7.73 for Amharic-Kistanigna and 4.43 for Kistanigna-Amharic translations, but highlighted the need for more parallel corpora.
Andargachew et al. (2023)	morpheme-based NMT	Investigated morpheme-based NMT models for low-resource fusion languages, demonstrating that morpheme-based models outperform conventional subword models on benchmark datasets, and created a new dataset for a low-resource language.

### 7.2 Identifying key challenges and limitations

Table 3 summarizes the key challenges and limitations faced by various research efforts in the field of Amharic-to-English machine translation, as documented in multiple studies from 2012 to 2023. The table is organized into two columns: Authors, and challenges and limitations.

**Table 3:** Summary the key challenges and limitations.

Author	Challenges and limitations
Teshome and Besacier (2012)	Limited computational linguistic resources and integrated linguistic knowledge. The unique Ge′ez-based writing system of Amharic complicates the adaptation of existing tools designed for languages with different scripts.
Teshome M. et al. (2015)	The linguistic diversity between Amharic and English presents significant challenges for machine translation (MT). Capturing nuances, idiomatic expressions, and cultural references is difficult due to the distinct characteristics of the two languages.
Abate et al. (2018) and Abate et al. (2019)	Due to the linguistic barrier, there is a shortage of data that hinders training of translation models. The existence of high structural differences within Ethiopian languages creates considerable issues for Statistical machine translation (SMT). Moreover, the scarcity of linguistics and NLP tools for African languages makes the problem even worse.
Gashaw and Shashirekha (2019, 2020)	Difficulties in domain adaptation, rare terms, lengthy sentences and phrases, as well as word alignment discrepancies are among the shortcomings of NMT. The absence of parallel corpora for the NMT Amharic-Arabic language pair, along with the rich morphology of Amharic, the lack of capitalization, and small sized machine-readable lexicons add to the complexity of the problem.
Mekonnen Gezmu et al. (2021)	Amharic-English machine translation poses a distinctive challenge due to morphological divergence of languages. NMT suffers heavily from the lack of data for effective training. Carefully designed implementation is required for subword-based models to outperform word-based models in translation. Linguistic translation tasks are further rendered difficult due to the divergence of linguistic syntactic structures.
Ashengo et al. (2021)	Large parallel corpora are essential for fluent machine translation, yet context unawareness in approaches like phrase-based machine translation (PBMT) hampers performance. While combinational approaches improve over simple NMT, rare words and uncommon vocabulary remain problematic.
Hadgu et al. (2022), Destaw Belay et al. (2022), and Negia et al. (2023)	The scarcity of datasets for low-resource languages limits the development of effective translation systems. Obtaining large-scale parallel corpora is challenging, and translation quality remains an issue for languages like Amharic. Additionally, limited Amharic linguistic resources and a small number of parallel sentences constrain deep learning experiments. Dependency on handcrafted features in rule-based MT further hampers progress.
Getachew and Yayeh (2023)	Dataset scarcity restricts extensive experimentation for improved results. Translating Ge′ez to English is time-consuming due to the script’s longer word counts and its agglutinative nature (i.e Amharic/Ge’ez languages combine multiple morphemes (word units) into single words, leading to longer and more complex expressions. This can make it difficult for translation models to accurately parse and understand the intended meaning), which adds to the computational complexity and increases training time for translation models. Additionally, Ge′ez is morphologically rich, meaning that words can take on several forms based on grammatical context. This richness can complicate encoding and require more sophisticated handling in machine translation task.
Gezmu (2023)	Variations in morphological typology present challenges in determining optimal vocabulary sizes for subword NMT models. Nondeterministic training processes and the lack of specified stopping criteria for NMT model training further complicate development.
Andargachew et al. (2023)	Neural machine translation requires substantial training data and parallel corpora. Ensuring faithful and fluent translations is challenging due to language variations. Recurrent neural networks (RNNs) struggle with long-distance dependencies, which are critical for accurate translations.

### 7.3 Assess current trends and future directions

Table 4 presents research trends and future directions in machine translation for Amharic and other low-resource languages on the basis of works published in 2022 and 2023. To assess current trends and future directions, we consider papers published in 2022 and 2023.

**Table 4:** Current trends and future directions.

Author	Current trends	Future directions
Gezmu and Nürnberger (2023), Biadgligne and Smaïli, 2022, and Gezmu (2023)	Corpus augmentation enhances MT models for under resourced languages. Token-level augmentation manipulates text to retain original semantics. Morpheme-based and subword-based NMT models outperform conventional models. Automated metrics such as BLEU and ROUGE offer a standardized and reproducible method for evaluating translation models, minimizing subjective biases that can arise in human assessments.	Investigate corpus augmentation impact on other under resourced language translations. Explore advanced tokenization techniques for further translation quality enhancement. Incorporate linguistic knowledge into NMT models for future research. Investigate efficacy of morphological segmentation tools in low-resource NMT. Explore morphological segmentation tools for low-resource NMT of fusion languages. Increase the size of the Amharic-English parallel corpus for NMT.
Hadgu et al. (2022)	Data preprocessing and model architecture of Lesan MT system contributed to its promising results for low-resource languages. Lesan outperforms Google Translate and Microsoft Translator in human evaluation. Lesan’s MT models are implemented using OpenNMT toolkit.	Leverage Lesan for broader language support on online platforms. Enhance Lesan’s translation model for more low-resource languages.
Destaw Belay et al. (2022) and Getachew and Yayeh (2023)	Transformer model dominates NMT paradigm for machine translation tasks. Normalization of Amharic homophones enhances Amharic-English machine translation performance. Limited studies on Amharic-English translation due to scarce linguistic resources. Ge′ez-English NMT using Transformer models shows promising results. Highly used evaluation metrics of machine translation is BLEU.	Expand dataset for more languages and use data augmentation techniques. Explore alternative pretrained language models for Amharic-to-English translation to serve as trainers, helping to address the challenges posed by low-resource settings. Use more corpora for higher quality results in future studies.
Gezmu et al. (2022) and Negia et al. (2023)	Extended parallel corpus for Amharic-English Machine Translation using SMT and NMT. Amharic-Kistanigna Bidirectional Machine Translation using Deep Learning.	Increase the size of the Amharic-English parallel corpus for NMT. Enhance bidirectional translation capabilities using advanced deep learning techniques.

## 8 Discussion

### 8.1 RQ1: what are the historical developments and milestones in the field of Amharic-to-English machine translation?

This systematic review revealed that the field of Amharic to English machine translation (MT) has undergone significant developments and milestones over the years, reflecting broader advancements in machine translation technologies. The following is a summary of the key historical developments and milestones:

*Early developments:* starting in 2012: Rule-based approaches (De Pauw et al., 2012; Kore and Goyal, 2017).

*Initial efforts:* the earliest attempts at machine translation between Amharic and English relied primarily on rule-based approaches. These systems were built via linguistic rules and require extensive knowledge of both languages’ grammar and syntax.

*Challenges:* these early systems faced challenges due to the complex morphology of Amharic and the lack of extensive digital resources.

*Statistical machine translation (SMT):* 2012 was the beginning of SMT for Amharic (Teshome and Besacier, 2012).

*Parallel corpora development:* the development of parallel corpora, such as the Amharic-English Bible corpus, provided essential data for training SMT models.

*GIZA++ and Moses toolkit:* tools such as GIZA++ for word alignment and the Moses toolkit for phrase-based SMT became instrumental in developing Amharic-English SMT systems.

*Notable works:* research projects and academic efforts during this period focused on leveraging SMT techniques, resulting in moderate improvements in translation quality using Ge’ez text (Tedla and Yamamoto, 2016; Teshome, 2013; Teshome and Besacier, 2012). These systems benefit from bilingual dictionaries and aligned texts, but their performance is still limited by the scarcity of large, high-quality parallel corpora.

*Neural machine translation (NMT):* 2019 was the beginning of the NMT for Amharic text (Gashaw and Shashirekha, 2019).

*LSTM and GRU models:* the introduction of neural machine translation models using long short-term memory (LSTM) and gated recurrent units (GRUs) marked a significant shift. These models were better at handling the complexities of Amharic grammar and provided improved translation accuracy compared with SMT (Biadgligne and Smaïli, 2022; Gezmu et al., 2022).

*Parallel corpus expansion:* efforts have been made to expand parallel corpora, incorporating news articles, government documents, and other bilingual texts to train more robust NMT systems (Gezmu et al., 2022).

*2022 - present: transformer models for the Amharic language* (Destaw Belay et al., 2022).

*Transformer architecture:* the adoption of transformer models, as exemplified by OpenNMT and similar frameworks, revolutionized the field. The transformers offered superior handling of long-range dependencies and contextual information, leading to substantial improvements in translation quality (Hadgu et al., 2022).

*Back-translation and data augmentation:* techniques such as back-translation, where monolingual Amharic texts are translated into English and then used to train the model, help mitigate the issue of limited parallel corpora (Biadgligne and Smaïli, 2022).

Overall, this development highlights the importance of technological innovation, custom-made methodologies and data enlargement in addressing the unique challenges of translating between Amharic and English. Future improvements in this domain will prospective focus on further refining these methods and expanding data resources to attain even greater translation accuracy and accessibility.

### 8.2 RQ2: what are the key challenges and limitations associated with past approaches to Amharic-to-English machine translation?

Throughout the years, Amharic to English machine translation (MT) has had its share of challenges, hurdles, and constraints. One of the major problems is deeply technological and linguistic in nature. Some of the challenges involve complex inflectional Amharic morphology which enables a word to encapsulate a great deal of grammatical information such as tense, aspect, person, gender, and number. These statistical and rule-based systems have a considerable amount of difficulty dealing with such complicated forms because their accuracy in parsing and generating complex forms is abysmal.

*Key challenges*

- Data scarcity

*Parallel corpora:* there is a significant lack of parallel corpora for Amharic and other Ethiopian languages, which is crucial for training both statistical and neural machine translation models. Although a number efforts have been made to develop parallel datasets for these languages (Abate et al., 2018; Destaw Belay et al., 2022; Gashaw and Shashirekha, 2020; Hadgu et al., 2022), the scarcity of such resources continues to hinder the development and improvement of translation systems. We therefore encourage further research and collaboration to address this gap and better support these low-resource languages.

*NLP resources:* the shortage of basic linguistic resources, such as morphological analyzers, machine-readable lexicons, and annotated datasets, impacts the effectiveness of translation models (Abate et al., 2018; Abate et al., 2019; Gashaw and Shashirekha, 2020; Gezmu et al., 2022).

The lack of parallel corpora and essential linguistic resources for Amharic and other Ethiopian languages hinders the advancement of machine translation systems drastically. The development of parallel texts which are both high quality and sufficient is needed for the training of statistical and neural machine translation models. Furthermore, none of these resources is available, such as annotated datasets and morphological analyzers, which makes the construction of reliable translation models even more difficult. There is no doubt that without overcoming these shortages, the use of machine translation in Amharic and other Ethiopian languages will continue to be ineffective and inadequate.

b. Morphological complexity

*Inflectional and derivational morphology:* Amharic’s rich morphological structure, where single words carry extensive grammatical information, poses a substantial challenge. The ability of language to create new words through various prefixes and suffixes increases the complexity of translation systems (Abate et al., 2019; Gashaw and Shashirekha, 2019; Gezmu, 2023; Mekonnen Gezmu et al., 2021).

*Agglutination:* the frequent combination of multiple morphemes into a single word adds another layer of difficulty for parsing and generating accurate translations (Gezmu, 2023).

c. Syntactic structure

*Word order:* the syntactic difference between Amharic’s subject-object-verb (SOV) order and English’s subject–verb-object (SVO) order necessitates complex reordering algorithms to maintain grammatical coherence and meaning during translation (Abate et al., 2018; Mekonnen Gezmu et al., 2021).

*Syntactic divergence:* the divergence in sentence structure requires advanced handling to preserve the intended meaning and fluency of the translation (Gezmu et al., 2021; Mekonnen Gezmu et al., 2021).

d. Domain mismatch and lexical issues

*Domain mismatch:* the challenge of translating domain-specific content due to differences in vocabulary and context between the source and target languages (Gashaw and Shashirekha, 2019).

*Rare words and long sentences:* the presence of rare words and long sentences further complicates the translation process, particularly in NMT systems (Ashengo et al., 2021; Gashaw and Shashirekha, 2019).

### 8.3 Limitations

- Unique writing system

*Capitalization and diacritics:* the absence of capitalization and the critical role of diacritics in Amharic add another layer of complexity to accurate translation (Gashaw and Shashirekha, 2019).

b. Handling nuances and cultural references

*Idiomatic expressions and proverbs:* Amharic has numerous idiomatic expressions and proverbs that do not have direct equivalents in English, requiring a deep understanding of the cultural context (Teshome M. G. et al., 2015).

*Honorifics and politeness:* variations in the use of honorifics and levels of politeness between Amharic and English necessitate careful handling to maintain appropriate tone and respect in translation (Teshome M. G. et al., 2015).

c. Technical constraints

*Training data requirements:* both statistical and neural machine translation models require large amounts of training data, which are challenging to obtain for low-resource languages such as Amharic (Abate et al., 2018; Ashengo et al., 2021; Biadgligne and Smaïli, 2022).

To sum up, doing Amharic-to-English machine translation is commendable, but accomplishing it is seemingly enveloped by insurmountable obstacles of inadequate technology, data, and the language’s intricacy. The lack of well-formed parallel corpuses and requisite NLP tools is greatly compounded by the rich fusional nature of Amharic as well as its comparatively more complex structural composition in relation to English which makes it almost impossible to create effective machine translation devices. In addition, American and Ethiopian cultural subtleties together with some specialized domains make it hard to improve the quality of the translation. Overcoming these problems calls for enhancing the current systems and resources by combining them with novel approaches, such as investing in deep text-to-text transform networks, particularly increasing the funding. Filling these voids will improve the process of translating Amharic to English making it accurate and more culturally relevant.

### 8.4 RQ3: what are the current trends and future directions in English-language machine translation research in Amharic?

Current research trends in Amharic to English machine translation focus on leveraging advanced NMT techniques, enhancing linguistic resources, and improving evaluation metrics. Future directions aim to expand these efforts by incorporating more sophisticated models, increasing data resources, and further refining translation techniques.

*Current trends*

1. Corpus augmentation and token-level manipulation

Researchers are focusing on corpus augmentation to enhance machine translation (MT) models for underresourced languages. Token-level augmentation, which manipulates text while retaining the original semantics, is particularly effective (Biadgligne and Smaïli, 2022; Gezmu, 2023).

Morpheme-based and subword-based neural machine translation (NMT) models are gaining traction, outperforming conventional subword models by leveraging automated evaluation metrics (Gezmu and Nürnberger, 2023; Gezmu, 2023).

2. Transformer models and normalization techniques

Transformer models dominate the NMT paradigm for machine translation tasks, especially for low-resource languages such as Amharic (Destaw Belay et al., 2022; Getachew and Yayeh, 2023). In addition, normalization techniques, such as addressing Amharic homophones, have been shown to enhance Amharic-English translation performance (Destaw Belay et al., 2022).

3. Extended parallel corpora and bidirectional translation

Research has focused on extending parallel corpora for Amharic-English machine translation, which is crucial for improving translation quality (Gezmu et al., 2022). Bidirectional translation models using deep learning techniques are being developed, showing promising results in improving translation accuracy (Negia et al., 2023).

### 8.5 Future directions

- Advanced corpus augmentation and tokenization

Future research should investigate the impact of corpus augmentation on other underresourced language translations and explore advanced tokenization techniques to further enhance translation quality (Biadgligne and Smaïli, 2022; Gezmu et al., 2022). The incorporation of linguistic knowledge into NMT models and improvements in morphological segmentation tools are suggested for advancing translation models (Gezmu et al., 2022).

b. Enhancing transformer models and data resources

Expanding datasets for more languages, using data augmentation techniques, and exploring other pretrained language models are crucial for improving Amharic-English translations (Destaw Belay et al., 2022; Getachew and Yayeh, 2023). The use of more corpora and enhancing translation quality through transformer models are essential next steps (Getachew and Yayeh, 2023).

For the most part, the latest analysis in the field of Amharic-English translation focuses on the application’s augmentation with modern neural machine translation (NMT) approaches such as adding transformer models, corpus enlargement, and manipulation at token levels. Moreover, parallel corpora extensions and the inclusion of cross-directional translation models have shown remarkable progress toward solving problems related to this language pair of limited resources. Further development of the translation system requires substantial improvement in tokenization techniques, data resource enhancement, and corpus expansion. Besides, the integration of pre-trained language models and the enrichment of the datasets with new topical areas need to be dealt with in order to advance the Amharic-English machine translation system capabilities.

## 9 Conclusion

The review of Amharic-to-English machine translation reveals a field in constant development and distinct difficulties. Researchers have attempted to automate the translation of Amharic, whose morphology and syntax are challenging due to their borders, utilizing their sophisticated and fluent irreducible finite state grammar by starting with basic, rule-based systems and progressing toward the currently prevalent, transformer-dominated systems. Although the application of tokenization techniques coupled with the usage of transformer architectures in neural machine translation (NMT) greatly enhances the performance in most cases, challenges such as lack of data, limited linguistic resources, and divergent syntax remain insurmountable obstacles. The transition from statistical to neural methods captures the evolution but also clearly draws attention toward the immense need of the hour in the form of adequate linguistic infrastructures and very creative solutions to deal with these intricacies in a sensible manner.

Amharic-English machine translation requires focused attention in specific domains in order to take further steps in developing the discipline. First, addressing the lack of data through more sophisticated augmentation approaches and widening the linguistic scope for building parallel corpora would make the models more robust. Second, utilizing accurate tokenization and segmentation of the Amharic language would improve the fidelity and accuracy of the translations. Moreover, linguistic integration in the neural machine translation architecture along with new low-resource language evaluation metrics will enable better benchmarking and comparison of model productivity.

Ultimately, working together across different areas to create detailed language resources and open access databases will help make the use of Amharic machine translation technologies more accessible and applicable in different fields. These initiatives are instrumental in moving the industry toward advanced, dependable, and context-sensitive translation services for Amharic and other less supported languages.

## Data Availability

The original contributions presented in the study are included in the article/supplementary material, further inquiries can be directed to the corresponding author.

## Footnotes

^1^ https://www.sciencedirect.com

^2^ https://scholar.google.com

^3^ https://www.acm.org

^4^ https://ieeexplore.ieee.org

^5^ https://www.springerlink.com
